# The 2025 Chikungunya Outbreak in Guangdong Province, China, and the Emerging Multi-pathogen Arboviral Threat in the Pearl River Delta

**DOI:** 10.7759/cureus.109439

**Published:** 2026-05-22

**Authors:** Elijah J Remis, Ali R Ahmed, Jack C Tietjen, Liyan Zhou, Jue Liu, Daniel R Lucey

**Affiliations:** 1 Medicine, Geisel School of Medicine at Dartmouth, Hanover, USA; 2 Epidemiology and Biostatistics, Peking University, Beijing, CHN

**Keywords:** aedes albopictus, arbovirus, chikungunya virus, chikv, climate, epidemic preparedness, guangdong, outbreak, pearl river delta, typhoon

## Abstract

Background

From mid-June into late December 2025, China experienced its largest-ever Chikungunya virus (CHIKV) outbreak across Guangdong Province, a densely populated region of southeast China with extensive international travel and established *Aedes* mosquito populations. CHIKV, dengue virus (DENV), and Zika virus (ZIKV) share the same *Aedes* vectors, overlap in clinical presentation, and exhibit serologic cross-reactivity. We describe the epidemiology and integrated public health response to this outbreak, as well as future arboviral risks and mitigation strategies.

Materials and methods

We synthesized weekly surveillance data from the Guangdong Provincial Center for Disease Control and Prevention, national epidemiological and genomic data from the China CDC and World Health Organization, Hong Kong Observatory climate records, published literature, and government bulletins. Outbreak dynamics were analyzed using confirmed-case counts disaggregated by prefecture-level city and reporting week.

Results

Guangdong reported over 25,800 locally transmitted CHIKV cases across all 21 prefecture-level cities, with nationwide cases exceeding 29,500. The outbreak occurred in two distinct waves: an initial wave centered on Foshan City (July-August; 8,948 cases) that was successfully contained through a rapid, multi-level public health response; and a second, larger wave in Jiangmen City (September-October; 10,035 cases) temporally coincident with three tropical cyclones that made landfall within a 16-day span, creating widespread *Aedes* breeding habitat. No deaths were reported in Guangdong. The causative lineage was the Middle African Lineage within the East/Central/South African genotype, carrying *Aedes albopictus*-adaptive mutations (E1-A226V, E2-L210Q, E2-I211T) independently acquired through convergent evolution with the Indian Ocean Lineage. The persistence of cases into mid-December, combined with the warmest winter on record in the Pearl River Delta, raises concerns about overwintering mosquitoes and an early resurgence in 2026.

Conclusions

The 2025 Guangdong outbreak suggests that extreme weather events can overwhelm even well-executed arboviral responses and that the climate-vector-arbovirus interface represents a systems-level vulnerability requiring anticipatory preparedness. We advocate for typhoon-landfall-triggered vector control deployment, intensified genomic and entomological surveillance beginning in spring 2026, multiplex molecular diagnostics for concurrent CHIKV/DENV/ZIKV detection, obstetric and neonatal arboviral screening, and cross-jurisdictional coordination across the Pearl River Delta and beyond.

## Introduction

Chikungunya virus (CHIKV) is a mosquito-borne alphavirus of the family Togaviridae that causes the clinical syndrome of Chikungunya fever. CHIKV is transmitted primarily by *Aedes albopictus* (*Ae. albopictus*) and *Aedes aegypti* (*Ae. aegypti*) mosquitoes [1,2]. The viral genome comprises a positive-sense, single-stranded RNA molecule of approximately 11.8 kb in length [2].

The pathogenesis of CHIKV infection follows a stepwise progression. When an infected *Aedes* mosquito bites a human and feeds on blood, it injects virus-containing saliva into the dermis, whereupon viral particles localize to dermal fibroblasts [1]. The virus binds to the MXRA8 receptor via the E2 envelope glycoprotein and enters cells by receptor-mediated endocytosis [1,3]. Viral replication proceeds in the host-cell cytoplasm [1].

After a mean incubation period of approximately three days, viral progeny gain access to the circulation. Resultant viremia causes an abrupt-onset fever that often exceeds 39°C and typically lasts seven days. Acute febrile illness is accompanied by polyarthralgia, myalgia, headache, and maculopapular rash [4]. Arthralgias can be debilitating and may persist or relapse for weeks or months after initial infection in up to 50% of patients. Some CHIKV infections remain asymptomatic. Clinical management is supportive; there is currently no approved antiviral therapy for CHIKV infection [1].

Early isolations of CHIKV were documented in Tanzania in 1952, in India in 1954, and in Thailand in 1958. Sporadic imported cases continued to be reported across Africa and Asia for the remainder of the 20th century. Over the last two decades, CHIKV has expanded beyond its historical endemic zones. The first large outbreak occurred in the Indian Ocean region on La Réunion Island in 2005-2006. Since then, outbreaks have occurred regularly across the globe, including in the Caribbean and the Americas (2013-2016); Brazil (2014, 2020-2024); Paraguay (2023); La Réunion (2009-2010, 2024); and China (2010 and 2025) [1,2,5].

Globalization and urbanization increase human mobility and population density, creating new opportunities for the redistribution of pathogens and vectors and increasing human-vector and vector-pathogen encounters. Human activities also alter the environment in which vectors propagate. For example, anthropogenic modifications to hydrology (e.g., land-use change, channelization, urbanization) can exacerbate flood severity, thereby creating breeding habitat for *Aedes* mosquitoes [6]. Concurrently, adaptive mutations to the E1 and E2 envelope glycoproteins have been shown to increase CHIKV transmission efficiency in *Ae. albopictus* mosquitoes [2,5].

In this study, our objectives were to (i) describe the temporal and geographic dynamics of the 2025 Guangdong Province CHIKV outbreak; (ii) examine the integrated public health and vector control responses across Foshan and Jiangmen; (iii) characterize the temporal association between tropical cyclone landfall and the later, larger Jiangmen outbreak (10,035 cases) relative to the initial Foshan epicenter (8,948 cases); and (iv) discuss implications for multi-arboviral preparedness during the 2026 *Aedes* season and beyond. Objectives (i)-(iii) are addressed descriptively using surveillance, genomic, and meteorological data; objective (iv) is a forward-looking synthesis informed by those data.

## Materials and methods

We synthesized epidemiological, genomic, entomological, and climate data about the 2025 CHIKV outbreak in Guangdong.

Epidemiological surveillance data

Weekly CHIKV surveillance reports published by the Guangdong Provincial Center for Disease Control and Prevention (Guangdong CDC) from 15 July 2025 through 29 December 2025 formed the primary epidemiological dataset [7]. These reports provided laboratory-confirmed case counts disaggregated by prefecture-level city and reporting week. Cases reported by the Guangdong CDC were classified as laboratory-confirmed CHIKV infections per the agency's standard surveillance reporting criteria; the specific laboratory diagnostic methods used to confirm the cases were not detailed in the publicly available weekly bulletins. National-level case data were obtained from the China Center for Disease Control and Prevention (China CDC Weekly) and the WHO Rapid Risk Assessment for Chikungunya (17 December 2025) [5,8].

Viral genomic data

Published whole-genome sequence analyses of 2025 Guangdong CHIKV isolates were reviewed to characterize the outbreak lineage and identify *Ae. albopictus*-adaptive mutations [2,9].

Climate and meteorological data

Tropical cyclone landfall records, sea-surface temperature observations for the South China Sea, and seasonal climate summaries were drawn from published Hong Kong Observatory reports covering the September 2025 outbreak-amplification period and the 2025-2026 winter, while the broader climate context was drawn from the Intergovernmental Panel on Climate Change's Sixth Assessment Report [10,11]. No primary meteorological data extraction or statistical reanalysis was performed.

Peer-reviewed literature

A non-systematic literature search was conducted in PubMed and Web of Science through 28 March 2026 using the terms "Chikungunya", "CHIKV", "Guangdong", "Pearl River Delta", "*Aedes albopictus*", "dengue co-circulation", "Zika", "arbovirus climate change", and "vector-borne disease preparedness". Reference lists of retrieved articles were hand-searched for additional relevant sources. No date restriction was applied, though preference was given to publications from 2020 onward.

Provincial and municipal bulletins

Official Chinese-language bulletins from the Guangdong provincial and Jiangmen municipal governments were accessed and translated to document the public health response timeline [7,12-15]. The Foshan municipal response was documented through published literature [8,13-15].

Analytic approach

Outbreak dynamics were characterized descriptively using confirmed-case counts by reporting week and prefecture-level city. The epidemic curve was plotted across the full reporting period (20 July-27 December 2025) to identify distinct transmission waves, peak incidence, and the temporal relationship between tropical cyclone landfall events and case surges. We did not perform lag regression, time-series, or other inferential modeling of the typhoon-case-surge association. Weekly case counts were compiled in Excel (Microsoft Corp., Redmond, WA, USA) and visualized in Python (pandas, matplotlib; Python Software Foundation, Wilmington, DE, USA). The administrative map was generated in Python using geopandas with administrative boundary data from the Database of Global Administrative Areas (GADM) version 4.1 (https://gadm.org). Viral genomic and entomological findings were interpreted in the context of previously published lineage characterizations, *Ae. albopictus*-adaptive mutation analyses and vector-ecology studies; the authors performed no primary sequencing or vector surveys.

## Results

2025 CHIKV outbreak, Guangdong, China

**Figure 1:**
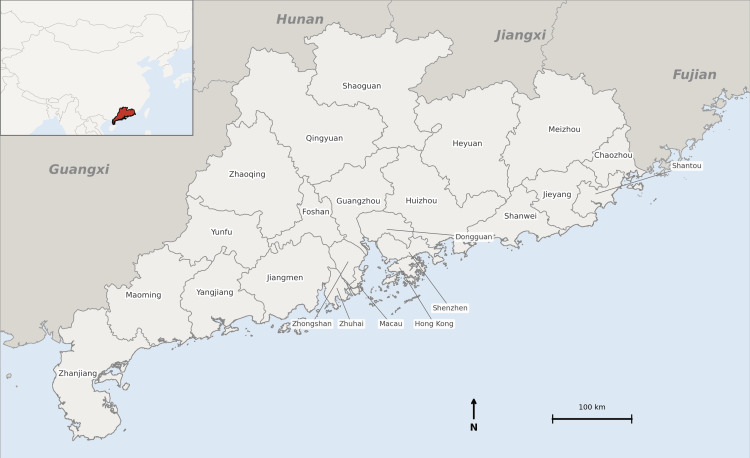
Administrative map of Guangdong, China, showing its 21 prefecture-level cities along with the Hong Kong and Macau Special Administrative Regions. The inset shows the location of Guangdong (red) within China The administrative map was generated in Python (Python Software Foundation, Wilmington, DE, USA) using geopandas with administrative boundary data from the Database of Global Administrative Areas (GADM) version 4.1 (https://gadm.org).

From mid-June to late December 2025, Guangdong experienced its largest-ever outbreak of CHIKV. The outbreak unfolded in two waves: an initial wave centered on Foshan City (July-August) and a second, larger wave in Jiangmen City (September-October), the latter temporally coincident with three tropical cyclones (Figure 1) [7].

Foshan City: initial epicenter and response

The first case was detected in the Shunde District of Foshan City, Guangdong, on 8 July 2025, though the earliest symptom-onset date was subsequently determined to be 16 June [8]. On 15 July, the Guangdong Center for Disease Control and Prevention began issuing daily situation reports via its official WeChat accounts and website, documenting 478 laboratory-confirmed cases that day [7]. Initial autochthonous transmission was concentrated in the neighborhoods of Lecong Town and Beijiao Town in Shunde District, Foshan City, but by 20 July 2025, cases had spread to adjacent areas [16]. Local health authorities began publishing weekly incidence reports; the first, covering 20-26 July and released on 27 July, documented 2,940 new laboratory-confirmed cases [7].

Foshan health officials activated a Level III public health emergency response on 29 July, the third-highest level under China’s four-tier national emergency plan [17]. The response integrated action at multiple administrative levels. The national government issued guidelines for diagnosis, treatment, and prevention and provided technical assistance [18]. The Guangdong provincial government coordinated logistical distribution of resources and personnel across Guangdong and compiled epidemiological data [7,18]. The Foshan municipal government oversaw operational command, expanded healthcare capacity, and deployed mosquito surveillance and vector control tools, including AI-enabled “electronic sentinels” and unmanned aerial vehicles (UAVs) [8,17-19]. District officials employed biological vector control (larvivorous fish and Wolbachia-infected mosquitoes) and promoted community participation in vector control [17].

**Figure 2:**
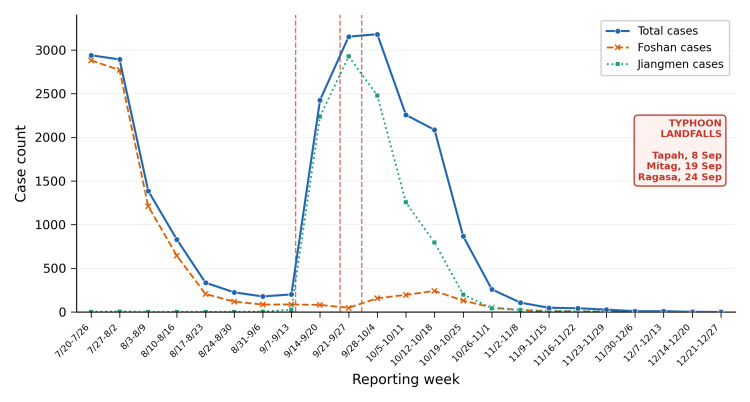
Weekly reported CHIKV case counts in Guangdong, 20 July-27 December 2025 Case data from the Guangdong Provincial Center for Disease Control and Prevention; tropical cyclone landfall dates from the Hong Kong Observatory. CHIKV: Chikungunya virus

Information bulletins advised residents on mosquito-control measures, including a district-wide mobilization weekend on 19-20 July [7]. From the reporting week of 27 July to 2 August onward, CHIKV cases in Guangdong declined markedly. The Level III public health emergency response in Foshan was terminated on 26 August, four weeks after activation, following nine consecutive days with fewer than 50 new CHIKV cases per day [17,18]. The total number of confirmed cases in 2025 in Foshan was 8,948 (Figure 2).

Jiangmen City: later epicenter and response

Despite successful containment in Foshan, three tropical cyclones (typhoons) made landfall in Guangdong in rapid succession during September 2025, preceding a CHIKV resurgence centered in Jiangmen City, approximately 35 km southwest of Foshan [20].

Severe tropical storm Tapah made landfall near Taishan in Jiangmen prefecture on 8 September; severe tropical storm Mitag made landfall near Shanwei City, 250 km northeast of Jiangmen, on 19 September; and super typhoon Ragasa, the most intense tropical cyclone to affect the South China Sea in 2025, struck near Yangjiang on 24 September, 150 km southwest of Jiangmen. Ragasa coincided with record-high sea surface temperatures in the South China Sea and a high spring tide, both of which compounded the severity of the storm surge across the Pearl River Estuary [20].

The Guangdong CDC weekly report for 7-13 September identified 201 new locally transmitted cases province-wide, with Foshan reporting 86 and Jiangmen 26. The following week (14-20 September), the province reported 2,426 new cases, a near hundredfold increase in Jiangmen alone, which reported 2,238 cases. Elevated case counts persisted over the subsequent two weeks, with 2,927 new locally transmitted CHIKV cases reported from 21-27 September and 2,480 from 28 September to 4 October (Figure 2) [7]. The total number of confirmed cases in Jiangmen in 2025, as reported by the Guangdong CDC weekly report, was 10,035 (Figure 2). Thus, the total number of confirmed cases reported in Jiangmen in 2025 exceeded that in Foshan (8,948) by 1,087 (12%).

Jiangmen City had reported its first locally acquired CHIKV case on 16 July; by 19 September, the cumulative count stood at 1,714, and the municipal government formally activated its Level III public health emergency response that day [12,13]. At a press conference on 20 September, the municipal government outlined a geographically tiered “three-circle” strategy targeting the epidemic site, the surrounding village or community, and the broader town or street level through systematic elimination of breeding sites, removal of stagnant water, and adulticiding. A mass-mobilization “Patriotic Health Campaign” promoted household-level mosquito control [13]. On 9 October, the Jiangmen Vector-borne Infectious Disease Prevention and Control Headquarters issued standardized technical guidelines for *Aedes* breeding-site management across the municipality [14]. By late October, the vector mosquito density compliance rate had exceeded 99% for 15 consecutive days; on 21 October, a joint national and provincial assessment concluded that sporadic low-level transmission had been achieved, and the Level III response was terminated on 24 October [15].

Province-wide spread and national scope

While the September epicenter was in Jiangmen, Guangdong CDC data confirm that CHIKV was spreading throughout the province by then. The province-wide outbreak reached its apex during the week of 28 September to 4 October: Guangdong reported 3,181 new locally transmitted Chikungunya cases, with no severe cases or deaths reported [7]. This was the first time since reporting began that laboratory-confirmed cases were identified simultaneously in all 21 prefecture-level cities. The report also identified laboratory-confirmed asymptomatic infections, though these were not quantified.

From the week of 5-11 October onward, new local cases declined steadily. However, new locally transmitted cases continued into mid-December, with 9 new cases reported for 7-13 December and 2 for 14-20 December. The report for the week ending 26 December, the final reporting week available, recorded zero new cases province-wide, the first such report since reporting began the week of 20-26 July 2025 [7].

According to the World Health Organization, as of 4 December 2025, a total of 29,497 locally confirmed cases had been reported in China (excluding Hong Kong Special Administrative Region (SAR), Macao SAR, and Taiwan) [5]. Guangdong accounted for 25,826 confirmed cases, approximately 88% of this national total (Figure 2).

Viral evolution and multi-pathogen risk

The 2025 Guangdong outbreak was caused by the Middle African Lineage (MAL), which is phylogenetically distinct from the Indian Ocean Lineage (IOL) that drove the 2005-2006 La Réunion epidemic. The Guangdong isolates shared greater than 99% genomic identity with MAL strains from the 2024-2025 La Réunion outbreak, consistent with a direct Africa-La Réunion-China importation pathway [2].

Notably, the Guangdong MAL strains harbor E1-A226V, E2-L210Q, and E2-I211T, the same *Ae. albopictus*-adaptive mutations previously associated with IOL. Their presence represents convergent evolution: the independent acquisition of an identical adaptive suite by two phylogenetically distinct ECSA sub-lineages under shared vector-selective pressure [9,21,22]. This convergence carries three implications. First, other ECSA sub-lineages could similarly adapt to *Ae. albopictus* and cause explosive outbreaks across the vector's range [21]. Second, geographic vulnerability is vector-dependent: regions with high *Ae. albopictus* density face elevated risk from ECSA-derived strains capable of acquiring E1-A226V [1,21,23]. Third, the sequential acquisition of second-step mutations E2-L210Q and E2-I211T atop E1-A226V further enhances transmission efficiency at the vector midgut barrier, suggesting that circulating strains may continue to optimize and that the 2025 outbreak may not represent the ceiling of *Ae. albopictus*-adapted CHIKV transmission potential [2,21,22].

Multi-pathogen arboviral co-circulation risk

The Pearl River Delta faces an elevated risk of concurrent or sequential arboviral transmission in 2026. The region experienced its largest dengue outbreak in 2014, with 37,382 cases in Guangzhou alone and a province-wide incidence of 47.3 per 100,000 [24]. A 2019 dengue virus (DENV) outbreak in Xishuangbanna Autonomous Prefecture, Yunnan Province, documented 12.62% CHIKV positivity among dengue-like patients and 68 cases of CHIKV coinfection among 697 confirmed DENV cases [25].

Zika virus (ZIKV) must also be considered. Southern China bears a disproportionate share of China's ZIKV importation exposure [26], and *Ae. albopictus* is a competent ZIKV vector [27]. Evidence of possible silent endemic circulation exists: 9.5% ZIKV IgG-positivity among individuals with no travel history in Guangdong’s neighbor, Guangxi [28]. A laboratory-confirmed DENV-ZIKV coinfection in China was detected on 19 January 2026 at a Sichuan port of entry in a traveler arriving from Kuala Lumpur [29].

CHIKV, DENV, and ZIKV share vectors, clinical presentations, and serologic cross-reactivity, creating diagnostic challenges that single-pathogen approaches cannot resolve. In co-circulation settings, the correct presumptive clinical diagnosis has been made in only 9-23% of laboratory-confirmed arboviral cases [30], reinforcing the need for multiplex diagnostic capacity at points of entry [29]. Vertical CHIKV transmission after maternal infection in late pregnancy can be associated with early neonatal neurological manifestations and a nearly twofold increase in adverse long-term neurodevelopment [31]. ZIKV poses a well-documented congenital risk, with congenital Zika syndrome occurring in 5-14% and microcephaly in 4-6% of exposed pregnancies, highest following first-trimester infection [32]. These obstetric considerations warrant integration into arboviral preparedness planning.

## Discussion

Experiences and lessons from the 2025 response

The 2025 Guangdong CHIKV outbreak offers an opportunity to evaluate the strengths and limits of a modern urban arboviral response in real time. The experience divides into what worked, what was overwhelmed, and what might be done differently in 2026.

Effective elements of the response

The initial response to the Foshan outbreak demonstrated that rapid, coordinated public health action can contain *Aedes*-borne transmission even in a densely populated urban environment. Within two weeks of the first situation report, authorities activated a multi-level response integrating national guideline issuance, provincial logistical coordination, and municipal operational command [8,17,18]. Tools deployed included UAV-based aerial spraying, AI-enabled vector surveillance, biological control, and community-based breeding-site elimination [17,18]. The Guangdong CDC’s weekly reporting system provided publicly accessible surveillance data enabling near-real-time tracking across all 21 prefecture-level cities [7]. The Level III emergency response in Foshan was activated on 29 July and terminated four weeks later, on 26 August, demonstrating successful containment [17,19]. No severe cases or deaths were reported in Guangdong throughout the outbreak [7,13].

Challenges exposed by the September typhoon sequence

The September typhoon sequence revealed that even a well-executed initial response can be overwhelmed by extreme meteorological events that exceed the capacity of vector control infrastructure. The resurgence centered on Jiangmen was, in several respects, more instructive than the earlier Foshan outbreak: new locally transmitted cases rose from 26 in the week of 7-13 September to 2,238 in the week of 14-20 September, constituting a near-hundredfold increase in a single week [7]. The speed of this escalation underscores that, even with excellent post-typhoon vector control mobilization, as in Jiangmen, it can be very challenging if not anticipated in terms of resources and a rapid, integrated response. The total number of confirmed cases reported by the Guangdong CDC in Jiangmen in 2025 was 10,035, approximately 12% higher than in Foshan (8,948) (Figure 2). This annual typhoon-related flooding risk may represent a systems-level vulnerability to *Aedes*-transmitted arboviruses such as CHIKV.

The biological pathway linking heavy rainfall to vector amplification is non-linear: intense rainfall can transiently flush larvae from container habitats, while expanded standing-water habitats post-storm provide oviposition sites and stimulate hatching of pre-existing eggs. The composite lag from any individual typhoon landfall to observed case count amplification reflects vector developmental dynamics, intrinsic and extrinsic incubation periods, and surveillance reporting delays; we did not model these formally. The temporal association between the September typhoon sequence and the Jiangmen case surge should therefore be interpreted as descriptive rather than causal.

Regional and global implications

The emergence of CHIKV in Guangdong in 2025 serves as a bellwether of shifting ecological and genomic paradigms in East Asia, the Mediterranean, Europe, and the Americas [33,34]. The outbreak did not remain geographically confined to the Pearl River Delta, and its implications extended beyond Guangdong's borders. Understanding those implications can be considered at three levels: within China, within East and Southeast Asia, and within the global trajectory.

Within China, CHIKV spread beyond Guangdong into nearby provinces. Confirmed local cases were reported in Guangxi (2,198 cases), Sichuan (358), Chongqing (296), Fujian (243), Hunan (146), and additional provinces, including Hainan and Jiangxi [5]. Several of these have established *Ae. albopictus* populations but limited prior CHIKV outbreak experience, meaning their populations are fully immunologically naïve. The 2026 risk profile in these provinces will depend heavily on whether locally transmitted chains were fully interrupted before winter, a question that the current surveillance data cannot definitively answer. Given the documented persistence of cases in Guangdong into mid-December and the overwintering capacity of *Ae. albopictus* eggs, the possibility of residual transmission foci being established in new provinces, including Guangdong, warrants active monitoring as the 2026 season approaches [35].

Guangdong's role as a sentinel for broader arboviral risk in East Asia has been explicitly articulated in the literature and is strongly reinforced by the 2025 experience [33]. For neighboring countries with similar *Aedes* ecology and comparable urbanization trajectories (Vietnam, Thailand, Malaysia, the Philippines, and Japan), the Guangdong 2025 outbreak serves as a concrete demonstration of what MAL-lineage CHIKV, carrying a full suite of *Ae. albopictus*-adaptive mutations, can produce in a susceptible urban population. Japan warrants particular attention: *Ae. albopictus* is well established across southern and central Honshu, urban population density is high, and the country's 2014 autochthonous dengue transmission established a precedent for a domestic *Aedes*-borne arboviral outbreak [36].

The 2025 Guangdong outbreak can be read as part of a trajectory of global CHIKV intensification. The World Health Organization reported 502,264 CHIKV cases globally in 2025, including 208,335 confirmed cases and 186 deaths [5]. These figures reflect a sustained two-decade expansion. The progressive adaptation of CHIKV to *Ae. albopictus* is a key mechanism facilitating geographic expansion, particularly in subtropical and temperate regions where *Ae. aegypti* is absent or sparse [33,37]. Europe and North America, where *Ae. albopictus* has expanded substantially over the past two decades, are not necessarily insulated from such CHIKV risk. The 2025 Guangdong experience provides a template for what importing an *Ae. albopictus*-adapted CHIKV strain into a naïve, vector-competent urban environment can produce.

Preparedness for the 2026 *Aedes*-arboviral season

The 2025 experience demonstrated that integrated vector control is effective when deployed rapidly and at scale, and that effectiveness is contingent on timing [17,18]. Emergency vector control stockpiles (larvicides, application equipment, and trained personnel) could be pre-staged in Pearl River Delta municipalities identified as hydrologically vulnerable, with deployment protocols triggered by typhoon landfall forecasts rather than confirmed case counts. Case counts may lag behind vector amplification. Investment in climate-resilient urban water-management infrastructure, including improved drainage capacity, flood-retention basin management, and reduction of low-lying stagnant-water zones across the Delta's urban districts, addresses the structural environmental conditions that vector control alone cannot overcome [6,38].

In contrast with *Ae. aegypti*, *Ae. albopictus* can undergo egg diapause, allowing survival through cold winter conditions [35]. The persistence of locally transmitted cases in Guangdong into mid-December raises concern that the overwintering period may be short enough for viremic humans to sustain low-level transmission that bridges the cold season and reignites the epidemic in spring 2026 [7]. According to the Hong Kong Observatory, December 2025 to February 2026 constituted the “warmest winter on record” in Hong Kong, with a mean temperature of 19.3°C, 2.0°C above average [39]. Entomological surveillance should be intensified in early spring 2026 to assess whether CHIKV-carrying *Ae. albopictus* eggs have overwintered, particularly in provinces that recorded local transmission late in the 2025 season [7].

Genomic surveillance capacity should be sufficient to characterize circulating strains in near-real time, enabling early detection of novel adaptive mutations before they drive outbreak amplification [2,9]. Systematic serosurveys in affected districts should be conducted and published to refine transmission models and to provide the population-level immunity estimates needed for credible risk assessment. Multiplex molecular diagnostics capable of simultaneously detecting all three arboviruses should be made widely accessible across primary care settings in Guangdong and other high-risk provinces as a frontline tool, not as a specialized reference laboratory capacity [29].

The scale of the immunologically naïve population across Guangdong and beyond means that the public health case for an effective, safe vaccine remains compelling [5]. Ixchiq (Valneva), a live-attenuated vaccine, received accelerated approval from the U.S. FDA in November 2023. However, postmarketing surveillance identified serious adverse events, including vaccine-strain encephalitis confirmed by CSF PCR, 21 hospitalizations, and three deaths; the FDA suspended the biologics license in August 2025, concluding that risks outweighed benefits [40]. Valneva subsequently withdrew its U.S. biologics license and investigational new drug applications in January 2026. Ixchiq remains approved in Canada, Europe, the United Kingdom, and Brazil, where a pilot vaccination program was initiated in January 2026 [41]. VIMKUNYA (Bavarian Nordic), a virus-like particle vaccine, is approved for use in the USA and Europe [42,43].

The multi-jurisdictional character of the 2025 outbreak, spanning 21 prefecture-level cities in Guangdong and involving at least six additional provinces, exposed the limits of response frameworks designed around single administrative units. Cross-provincial coordination protocols, including pre-agreed data-sharing arrangements, joint surveillance reporting, and coordinated vector control mobilization across administrative boundaries, should be developed and exercised before they are needed. The Africa-La Réunion-China importation pathway that seeded the 2025 outbreak illustrates that arboviral preparedness in Guangdong should be linked with arboviral surveillance in the Indian Ocean region and sub-Saharan Africa. Bilateral and multilateral information-sharing mechanisms with regional partners should be strengthened accordingly [5,33].

Strengths

This analysis has several strengths. First, it integrates weekly surveillance, genomic, climate, and government-bulletin sources into a single coherent descriptive narrative spanning the full 2025 outbreak across all 21 prefecture-level cities in Guangdong. Second, primary-source Chinese-language provincial and municipal bulletins were translated by the authors, providing English-language access to operational details of the Guangdong, Foshan, and Jiangmen public health responses, including both the successful Foshan containment and the subsequent limits of that response when challenged by extreme weather in Jiangmen. Third, the analysis translates the 2025 experience into specific operational recommendations that may inform preparedness planning in subsequent *Aedes*-arboviral seasons across Guangdong, the broader Pearl River Delta, and other jurisdictions with comparable vector ecology: pre-staged vector control stockpiles triggered by typhoon-landfall forecasts, multiplex CHIKV/DENV/ZIKV diagnostics, intensified entomological and genomic surveillance, and cross-jurisdictional coordination.

Limitations

Several limitations should be noted. First, despite the comprehensive weekly epidemiological data from Guangdong CDC surveillance reports and periodic WHO situation assessments [5,7], reporting delays, variation in testing capacity across prefecture-level cities, and under-ascertainment of mild and asymptomatic infections may bias the observed epidemic curve. The documented presence of asymptomatic laboratory-confirmed cases suggests the true attack rate may be higher than confirmed case counts indicate. Still, the magnitude of under-ascertainment cannot be determined without population-based seroprevalence data. Second, the climate and meteorological data rely primarily on Hong Kong Observatory records as a proxy for Pearl River Delta conditions; temperature, precipitation, and humidity may differ materially in inland Guangdong cities. Third, our analysis is descriptive rather than inferential. We did not perform lag regression, time-series, or other quantitative modeling of the relationship between typhoon landfall and case count amplification, and the temporal association reported should be interpreted accordingly rather than as a causal demonstration. Fourth, genomic and entomological interpretations rely on published literature rather than primary sequencing or vector surveys conducted by the authors. Fifth, the 2026 projections presented are scenario-based assessments drawing on climate forecasts, vector-ecology models, and assumptions about viral evolution and human behavioral responses. They should be interpreted as such rather than as predictions. Sixth, the literature component of this analysis was a narrative synthesis rather than a systematic review and was therefore subject to selection bias; we did not apply PRISMA screening, dual independent extraction, or pre-registered inclusion criteria, and did not maintain records of database-specific search strings or screening counts. Seventh, the absence of population-based seroprevalence data precludes estimation of the true attack rate or population-level immunity in affected districts.

## Conclusions

The two-wave pattern, a containable Foshan epidemic followed by a Jiangmen resurgence temporally associated with sequential typhoon landfalls, highlights the need for pre-staged vector control deployment triggered by typhoon-landfall forecasts. Case persistence into mid-December warrants intensified entomological and genomic surveillance and cross-jurisdictional coordination. The co-circulation risk of CHIKV, DENV, and ZIKV supports the integration of multiplex diagnostics and obstetric arboviral screening into preparedness frameworks, alongside cross-jurisdictional coordination across Guangdong.
